# Plexin-B1 plays a redundant role during mouse development and in tumour angiogenesis

**DOI:** 10.1186/1471-213X-7-55

**Published:** 2007-05-22

**Authors:** Pietro Fazzari, Junia Penachioni, Sara Gianola, Ferdinando Rossi, Britta J Eickholt, Flavio Maina, Lena Alexopoulou, Antonino Sottile, Paolo Maria Comoglio, Richard A Flavell, Luca Tamagnone

**Affiliations:** 1 Institute for Cancer Research and Treatment (IRCC), University of Torino Medical School, Division of Molecular Oncology, Candiolo, Turin 10060, Italy; 2 Department of Neuroscience, Rita Levi Montalcini Centre for Brain Repair, University of Turin, I-10125 Turin, Italy; 3 MRC Centre for Developmental Neurobiology, King's College London, Guy's Campus, London, SE1 1UL, UK; 4 Developmental Biology Institute of Marseille – Luminy (IBDML) UMR 6216, CNRS – Université de la Méditerrannée, Campus de Luminy – Case 907, France; 5 Laboratory of Clinical Biochemistry, University of Torino Medical School, I-10060 Candiolo (Torino), Italy; 6 Department of Immunibiology, Yale University School of Medicine and Howard Hughes Medical Institute, New Haven, CT, USA; 7 Centre d'Immunologie de Marseille-Luminy, CNRS-INSERM- Université de la Méditerranée, 13288 Marseille, France

## Abstract

**Background:**

Plexins are a large family of transmembrane receptors for the Semaphorins, known for their role in the assembly of neural circuitry. More recently, Plexins have been implicated in diverse biological functions, including vascular growth, epithelial tissue morphogenesis and tumour development. In particular, PlexinB1, the receptor for Sema4D, has been suggested to play a role in neural development and in tumour angiogenesis, based on in vitro studies. However, the tissue distribution of PlexinB1 has not been extensively studied and the functional relevance of this receptor in vivo still awaits experimental testing. In order to shed light on PlexinB1 function in vivo, we therefore undertook the genomic targeting of the mouse gene to obtain loss of function mutants.

**Results:**

This study shows that PlexinB1 receptor and its putative ligand, Sema4D, have a selective distribution in nervous and epithelial tissues during development and in the adult. PlexinB1 and Sema4D show largely complementary cell distribution in tissues, consistent with the idea that PlexinB1 acts as the receptor for Sema4D in vivo. Interestingly, PlexinB1 is also expressed in certain tissues in the absence of Sema4D, suggesting Sema4D independent activities. High expression of PlexinB1 was found in lung, kidney, liver and cerebellum.

Mutant mice lacking expression of semaphorin receptor PlexinB1 are viable and fertile. Although the axon collapsing activity of Sema4D is impaired in PlexinB1 deficient neurons, we could not detect major defects in development, or in adult histology and basic functional parameters of tissues expressing PlexinB1. Moreover, in the absence of PlexinB1 the angiogenic response induced by orthotopically implanted tumours was not affected, suggesting that the expression of this semaphorin receptor in endothelial cells is redundant.

**Conclusion:**

Our expression analysis suggests a multifaceted role of PlexinB1 during mouse development and tissue homeostasis in the adult. Nonetheless, the genetic deletion of PlexinB1 does not result in major developmental defects or clear functional abnormalities. We infer that PlexinB1 plays a redundant role in mouse development and it is not strictly required for tumour induced angiogenesis.

## Background

Plexins are a highly conserved family of single pass transmembrane receptors which, in mammals, comprises nine genes grouped into four subfamilies (A thru D) based on sequence homology [1]. They are characterized by a conserved sequence, the "sema domain", a structural domain that mediates protein-protein interaction, and phylogenetically links the Plexins to the Semaphorins and the Scatter Factor Receptors [2]. The intracellular domain is highly conserved among the Plexins but does not share striking homology with other known proteins. Although the mechanisms of Plexin-mediated signalling have not been well understood, they are known to impinge on cytoskeletal dynamics and on cell adhesion, e.g. by regulating monomeric GTPases of Rho and Ras families [3].

Plexins were initially characterized for their role in axon guidance, where they function, either alone or in complex with the Neuropilins, as semaphorin receptors. Subsequently, Plexins were shown to control immune response and angiogenesis, and more recently were proposed to orchestrate tissue morphogenesis and cancer progression [4]. We have previously identified PlexinB1 as the receptor for Sema4D. Upon ligand binding, PlexinB1 regulates cytoskeletal remodeling (via Rac [5,6], PDZ-RhoGEF [7], p190 RhoGAP [8]), integrin activation (via R-Ras [9], PI3K [10]), MAPK signalling [11], cytosolic tyrosine kinases (Src, PYK2) [12], and can trigger the activation of the tyrosine kinase receptors Met and ErbB-2 [13,14].

It was also demonstrated that PlexinB1 can mediate axon outgrowth [15] and endothelial cell migration [16]. In addition, its ligand Sema4D is a potent angiogenetic factor [16,17], possibly involved in tumour induced angiogenesis [18].

PlexinB1 mRNA expression has previously been shown in the nervous system [19] and in diverse tissues at E14 of murine development [20]; on the other hand, detailed information on the expression of Sema4D mRNA outside the nervous system is missing. Notably, PlexinB1 and Sema4D expression at protein level by immunohistochemistry has not been reported yet. Furthermore, little is known about the expression of Sema4D and PlexinB1 in the adult tissues.

In spite of extensive studies performed on Plexins of A subfamily, molecular genetic studies to address the functional relevance in vivo of Plexins-B are currently lacking. Here we report our analysis of PlexinB1 deficient mice, including functional studies to test the relevance of this semaphorin receptor in tumour induced angiogenesis.

## Results

### PlexinB1 and Sema4D expression during embryo development and in the adult

**Figure 1:**
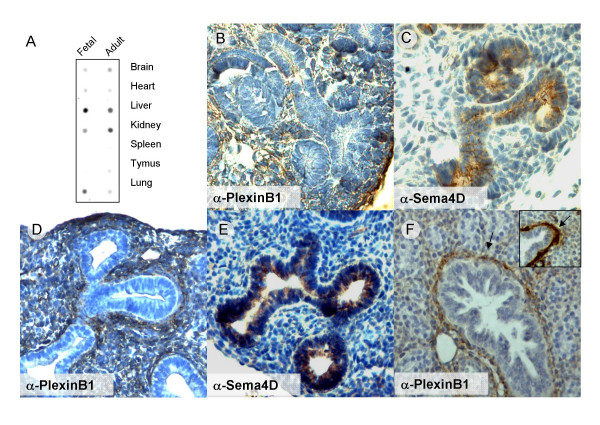
**Expression of PlexinB1 and Sema4D in in different organs and during branching morphogenesis**. A) PlexinB1 expression in different human organs was assessed by mRNA Dot Blot analysis. In the mouse embryo at E14 PlexinB1 is present in the mesenchimal cells of kidney (B) and lung (D) while Sema4D displays a complementery expression pattern in the epithelial cells of the same organs (C and E respectively). F) At E17 PlexinB1 expression in the lung is found in the mesenchymal cells positive for Smooth Muscle Actin (arrow). The inset shows the anti-Smooth Muscle Actin staining, for comparison.

In order to get insights to their physiological function, we investigated the tissue distributions of PlexinB1 and Sema4D in embryonic and adult tissues. Initially, we explored PlexinB1 mRNA expression by Dot Blot analysis of human tissue (Figure 1A) and by whole mount In Situ Hybridization of mouse embryos (Additional file 1). Taken together, these data revealed a wide expression of PlexinB1 in various tissues, including nervous system, lung, kidney and liver.

In order to gain further evidence of PlexinB1 expression at the protein level, we generated specific anti-PlexinB1 antibodies (see Additional file 2 for specificity controls), which we used to analyze further embryonic and adult tissues.

The immunohistochemical analysis of PlexinB1 and Sema4D expression revealed an intriguing distribution in developing organs characterized by epithelial branching morphogenesis, including the kidney, the lung and the pancreas. During epithelial branching morphogenesis, tissues are shaped by the outgrowth and branching of tubular epithelial structures, which invade the surrounding mesenchyme under the control of growth factors and guidance cues. We found that, at an early stage, PlexinB1 is expressed in the mesenchyme surrounding epithelial tubules in kidney and lung (Figure 1B, D) and in the pancreas (not shown), while Sema4D is expressed by epithelial cells (Figure 1C, E).

**Figure 2:**
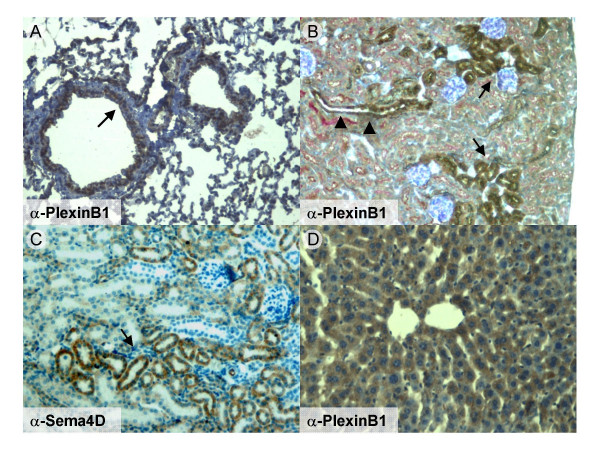
**PlexinB1 and Sema4D are expressed in different epithelial tissues in the adult mouse**. A) PlexinB1 is expressed in the epithelium of the lung airways.(arrow). B) In the kidney PlexinB1 staining is present in the distal tubules (arrow) and the collecting ducts (arrowhead); the proximal tubules are stained in red by PAS histochemical reaction. C) In the kidney, Sema4D is mainly expressed in the pars convoluta of the proximal tubules. D) In the adult liver PlexinB1 is diffusely expressed in the liver parenchima.

The developmental expression of this ligand-receptor pair appears to be dynamically regulated. For instance, in the lung at E17, PlexinB1 was expressed in cells of putative smooth muscle lineage recruited to wrap the differentiating airways (Figure 1F); conversely, in the adult, PlexinB1 expression was mostly localized to bronchial epithelium (Figure 2A). Notably, while Sema4D expression was clear in developing epithelia (Figure 1C), it was undetectable in the adult lung (not shown).

In the adult kidney, we found an elevated expression of PlexinB1 in distal tubules and collecting ducts (Figure 2B), while Sema4D displays a complementary expression in the pars convoluta of proximal tubules (Figure 2C).

In the mouse liver, we could not clearly detect PlexinB1 expression during early embryonic stages (not shown), though we found a high expression of PlexinB1 in adult hepatocytes (Figure 2D). Surprisingly, we could not detect Sema4D expression in the liver at any stage (not shown), suggesting Sema4D-independent activities of PlexinB1 in this tissue.

**Figure 3:**
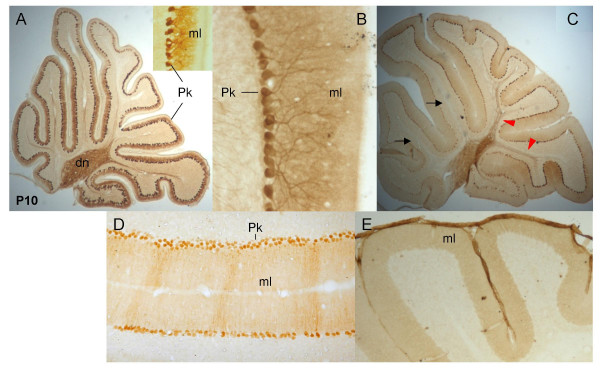
**PlexinB1 and Sema4D expression in developing and adult cerebellum**. P10 (A) and adult (B-E) cerebellum. In A, B, C, E left is rostral, right is caudal. A) In the P10 cerebellum PlexinB1 is homogeneously expressed in Pk cell perikarya, axons (A) and in the dendrites invading the molecular layer (inset). B) This expression is maintained in adult Pk cells. At this age, however, labelling intensity of Pk axons shows a clear rostrocaudal gradient (compare the black arrows with the red arrowheads in C). In addition, frontal sections reveal different expression levels in parasagittally-oriented Pk subsets (D). E) Diffuse Sema4D immunoreactivity is present in the adult molecular layer. Pk, Purkinje cells; dn, deep nuclei; ml, molecular layer.

Consistent with previous reports [19], in the nervous system we detected PlexinB1 expression in sensory and motor neurons, but were particularly attracted by the distinctive expression of PlexinB1 and Sema4D during cerebellar development. During postnatal development, Purkinje cell (Pk) dendrites expand into the nascent molecular layer, to form extensive connections with granule cell axons (parallel fibres). In the P10 cerebellum, PlexinB1 is exclusively expressed in Purkinje cells, being distributed in the perikaryon, dendritic tree and axon, up to the terminal branches in the deep cerebellar nuclei (Figure 3A). At the same developmental stage it was shown that Sema4D is expressed by myelinating oligodendrocytes in the folial white matter [21]. In the mature cerebellum PlexinB1 expression remains selectively confined to PK cells. Moreover, labelling intensity of Pk axons now reveals a clear cut rostro-caudal gradient, with higher levels of expression in the posterior cortical lobules (Figures 3B, C). In addition, analysis of frontal sections shows differential expression along parasagittally oriented Pk cell subpopulations (Figure 3D). At this age, anti-Sema4D antibodies show a diffuse distribution pattern in the molecular layer, suggesting expression in parallel fibres (Figure 3E).

### Generation of PlexinB1 null mutant

**Figure 4:**
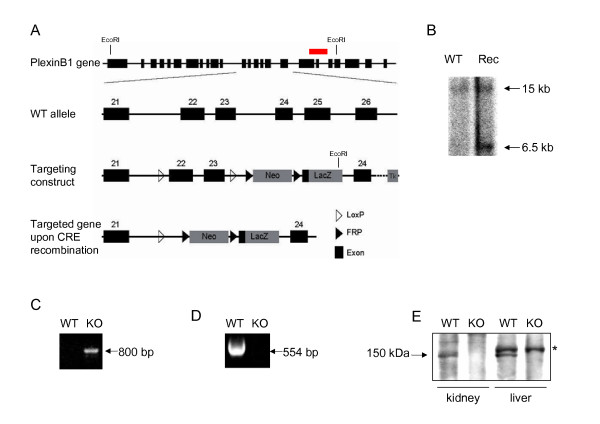
**Generation of PlexinB1 mutant mice**. A) Schematic structure of murine PlexinB1 gene, targeting construct and recombinant allele upon CRE deletion. In red is indicated the position of the probe for the Southern blot. B) Southern blot analysis of genomic DNA from recombinant ES cells after EcoRI digestion. A 15 Kb band in the WT and a 6.5 Kb band in the homologous recombinant (EcoRI sites are shown in A). C) The excision of exons 22 and 23 was checked using a primer pair that gives an amplicon of 800 bp upon CRE deletion and does not amplify the WT gene. D) RT PCR to reveal PlexinB1 mRNA was performed on total RNA from adult kidney. E) PlexinB1 protein in mutant mice is undetectable by IP and Western blot analysis of adult kidney lisates; the asterisk indicates a non specific band recognized by the antibody in the liver only.

To investigate the functional relevance of PlexinB1 in vivo, we undertook the genomic targeting of the murine gene to obtain PlexinB1-deficient mice. To this end, we generated a targeting construct in which we placed exon 22 and 23 of the PlexinB1 gene (encoding the transmembrane domain of the receptor) between two loxP sites (Figure 4A). Furthermore, we inserted a Neo cassette, flanked by two FRP sites and followed by a LacZ reporter fused to the first exon of the intracellular portion of the PlexinB1 gene; this allowed us, upon FLP recombination, to also obtain knock-in PlexinB1 mutants. The correct targeting of the construct in the genome of ES cells was confirmed by Southern blot analysis (Figure 4B). Chimeric mice, obtained from a positive ES cell clone, were mated with a CRE deleter strain to achieve gene inactivation, and eventually backcrossed into C57BL/6 genetic background. The actual deletion of exons 22 and 23 in mouse genome, encoding for the transmembrane region of PlexinB1, was confirmed by PCR analysis (Figure 4C).

By crossing heterozygous mice, we obtained PlexinB1 homozygous mutant mice, which were born and developed normally into adulthood. The genotypic analysis revealed the expected Mendelian ratios in littermates (Table 1). We confirmed the absence of PlexnB1 mRNA in homozygous mutant by RT PCR analysis (Figure 4D). Moreover, we analyzed tissue extracts of mutant and control mice by Western blotting by means of an antibody directed to the intracellular domain of the receptor, and confirmed that the expression of PlexinB1 was abrogated in homozygous mutant mice at the protein level (Figure 4E).

**Table 1:** Mendelian distribution of PlexinB1 deficient mice in offsprings

	No.	%
**Wild type**	31	25.0
**Heterozygous**	63	50.8
**Homozygous**	30	24.2

The genotyping of littermates born from heterozygous × heterozygous breeding pairs showed the expected mendelian ratio.

### PlexinB1 deficient mice do not display major developmental defects

PlexinB1 mutant mice did not show any detectable difference in size and were undistinguishable from their wild type (WT) littermates; in addition, their feeding behaviour and weaning appeared to be regular, they developed normally and did not show defects in reproduction or fertility. Moreover, we measured some of the main blood parameters (i.e. white blood cells counts, red blood cells counts and values, haemoglobin concentration, platelet count) and found them to be normal (data not shown). Thus we undertook a histological analysis of embryonic and adult tissues in PlexinB1 null mice.

**Figure 5:**
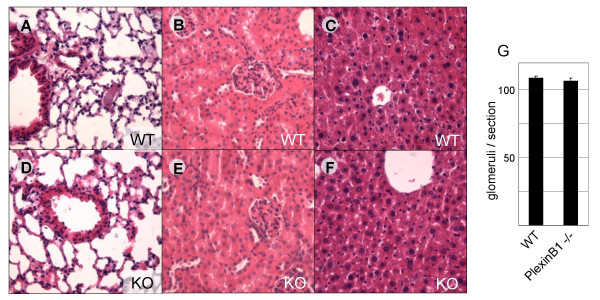
**Histology of lung, kidney and liver is normal in PlexinB1 mutant mice**. Hematoxilin & Eosin staining of WT tissue sections (A, B, C) and Knock-out (D, E, F). The analysis of lung (A, D), kidney (B, E) and liver (C, F) sections from WT and mutant animals did not reveal any major abnormality in the histology of these organs. Moreover, the number of glomeruli per section was equal in WT and in the PlexinB1 deficient mice (G).

Lung development provides a prototypic example of branching morphogenesis and semaphorins and plexins were shown to play a role during this morphogenetic process [22]. However, upon histological examination we failed to reveal any obvious abnormality in the morphology of lung epithelium or in the septation of the airways in PlexinB1 null mice (compare Figure 5A to 5D). Besides, the alveoli were properly inflated and the respiration occurred normally in all the animals analyzed.

In the kidney PlexinB1 is highly expressed; however, the histological analysis of mutant mice did not reveal any gross defect or abnormality in tubules or nephrons (compare Figure 5B to 5E). These specialized structures develop as the result of an intricate dialogue between the epithelial cells and the surrounding mesenchyme, and these results implicate that PlexinB1 is not essential during nephrogenic induction. Furthermore, the number of renal glumeruli were counted and found to be similar in WT and mutant mice (Figure 5G). Because significant defects in kidney function may not implicate gross histological abnormalities and because PlexinB1 is also expressed in the adult, we performed blood tests on two major indicators of renal function, creatinine and urea levels, which turned out to be comparable in normal and mutant mice (see Tab. 2).

We furthermore examined the liver parenchyma, where PlexinB1 is strongly expressed in WT mice. The haematoxylin/eosin staining revealed no sign of fibrosis, steatosis or other particular abnormality in mutant mice (compare Figure 5C to 5F). We performed blood tests to check two major indicators of liver function: i.e. plasma concentration of fibrinogen and total proteins. In addition, we measured blood levels of hepatic transaminases (glutamic oxalacetic transaminase and glutamic piruvic transaminase), which are elevated in the presence of liver disorders, and we assessed the integrity of biliar tree by measuring the blood levels of gamma-glutamil-transferase and alkaline phosphatase. None of these analyses revealed significant differences between WT and mutant mice (Table 2).

**Table 2:** Blood tests

	**UREA**	SD	**CREA**	SD	**GOT**	SD	**GPT**	SD
	
	mg/dl		mg/dl		U/l		U/l	
**WT**	11.0	± 1.0	0.167	± 0.025	42.3	± 0.57	23.7	± 2.08
**PlexinB1 KO**	11.8	± 1.7	0.153	± 0.026	42.3	± 3.40	22.8	± 1.89
								
	**GGT**	SD	**ALP**	SD	**FIB**	SD	**TP**	SD
	
	U/l		U/l		mg/dl		gr/dl	

**WT**	7.67	± 1.52	127	± 6.80	184	± 5.1	3.17	± 0.035
**PlexinB1 KO**	7.25	± 1.89	131	± 5.68	186	± 3.5	3.26	± 0.27

CREA: creatinine

GOT: glutamic oxalacetic transaminase

GPT: glutamic piruvic transaminase

GGT: gamma-Glutamil-Transferase

ALP: alkaline phosphatase

FIB: fibrinogen

TP: total protein

**Figure 6:**
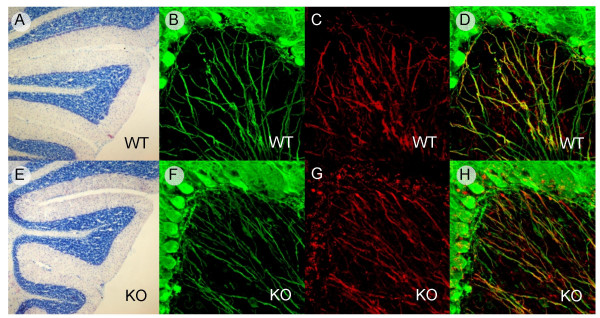
**Purkinje cell axons are correctly my elinated in PlexinB1 -/- mice**. Cerebellar histology and myelination of Pk axons in WT (A to D) and PlexinB1 deficient mice (F to H). Cerebellar histology is undistiguishable in WT (A) vs mutant mice (E) and the foliation of cerebellar lobuli occurs normally. Anti Calbindin (green) staining shows that Pk axons develop nomally in PlexinB1 mutant and they are correctly myelinated (as shown by anti MBP immunoreactivity in red). The merge is shown in D and H.

As mentioned above, we found the highest PlexinB1 protein expression in the central nervous system within the cerebellum. However, in PlexinB1 null mice, the overall cerebellar morphology of the cerebellum appeared normal and we could not reveal any abnormality in the foliation of lobuli or in the number of Pk cells (Figure 6A, E). In the cerebellum, myelin formation starts from the first postnatal week, in a period in which Sema4D is expressed by the olygodendrocytes [21]. This prompted us to scrutinize the myelination process of Pk axons by means of confocal analysis. In fact, anti-myelin basic protein (MBP) staining showed a correct wrapping of Pk axons by the glial cells (Figures 6B, C, D, F, G, and 6H). In addition, Pk axons were immunolabeled with anti-Calbindin (a major Pk cells marker) in the mutant mice, revealing a normal axon patterning. Furthermore, the collateral branching of Pk axons in the infraganglionic plexus, which is normally confined in the most superficial portion of the granular layer [23], did not show any abnormality (see Figure 6B, F). Also, the dendritic arborization of Pk cell in the molecular layer appeared normal in the mutant mice (not shown). These data suggest that, although PlexinB1 is abundantly expressed in Pk cells, its function doesn't seem to be essential for proper development of cerebellar Pk cells.

### Axonal growth cones from PlexinB1 mutants fail to collapse in response to Sema4D

**Figure 7:**
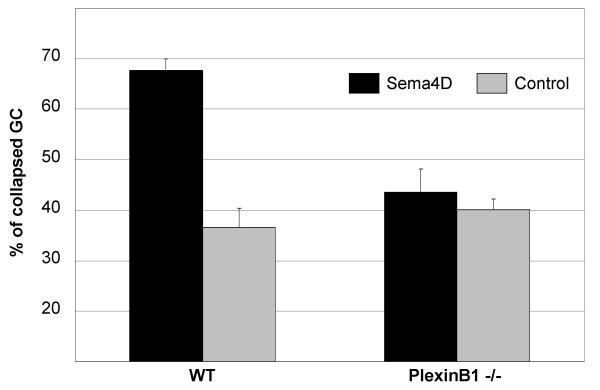
**PlexinB1 -/- hippocampal neurons do not respond to Sema4D**. Hippocampal neurons were stimulated with Sema4D for 1 hour and the percentage of growth cones (GC) positive axons was counted.

We thought to exploit these mutants as a genetic tool to investigate the functional requirement of PlexinB1 in assays previously used to study Sema4D signalling. For instance, it was demonstrated that the axonal growth cones of cultured hippocampal neurons undergo collapse in response to Sema4D in vitro, and it was proposed that this response is mediated by PlexinB1 receptor [9]. We observed that PlexinB1 is expressed by hippocampal neurons, although at a lower level compared to Pk cells (not shown); however, the evidence that PlexinB1 is responsible for the transduction of Sema4D repulsive signals in these neurons is still lacking. Thus, to verify whether this functional response is abrogated in PlexinB1 deficient mice, hippocampal neurons derived from WT or mutant mice were cultured for 48 hours ex vivo and then incubated with Sema4D for 1 hour. While the number of collapsed growth cones in wild type neurons was strongly increased upon Sema4D stimulation, PlexinB1 -/- hippocampal neurons did not collapse and underwent normal axonal outgrowth and differentiation, irrespective of Sema4D treatment (Figure 7). This establishes a functional requirement for PlexinB1 in the repelling activity of Sema4D in hippocampal neurons. We then examined the overall histology and the cellular composition of the hippocampus in PlexinB1 knock-out mice. Moreover, we analysed the cytoarchitectonic and the connection pattern of hippocampal neurons (stained for Calbindin marker) and interneurons (immunolabeled with anti-Parvalbumin antibody). However, we did not observe obvious abnormalities of this neural structure in mutant mice (see Additional file 3), consistent with a compensation of Sema4D function in the development of the hippocampus.

### PlexinB1 is dispensable for tumour-induced angiogenesis

Sema4D is a potent angiogenetic factor in vivo [16,17] and it is released by many tumour cells [[18]; and our unpublished data]. Furthermore, it was shown that inhibiting the expression of Sema4D in human head and neck carcinoma cells leads to reduction of tumour burden and vascularity in a mouse xenograft model [18].

In fact, the so called "angiogenic switch", i.e. the ability of tumour cells to recruit new vessels in order to provide blood supply, is a basic step in solid tumour growth and metastatic dissemination. Without overcoming this rate-limiting step, most tumours cannot grow over 2 mm in diameter, nor metastasize. PlexinB1 is known to transduce Sema4D signals in vitro and it is expressed in HUVEC endothelial cells and in the tumour vessels in vivo [16-18], however its requirement in tumour angiogenesis has not been clearly established.

**Figure 8:**
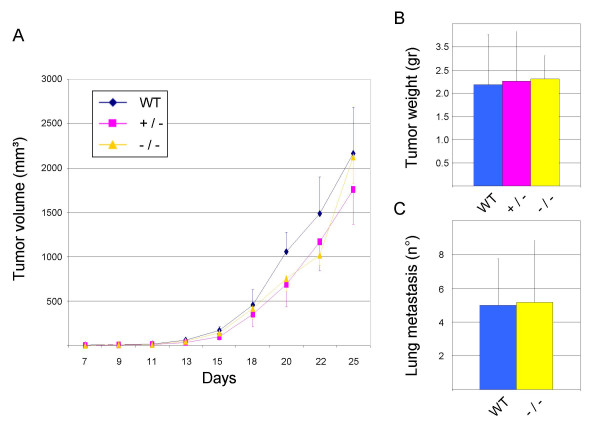
**Tumour growth in PlexinB1 mutant mice**. A) B16 melanoma cells were injected orthotopically in WT, heterozygous and homozygous PlexinB1 deficient mice and tumour volume was measured over time. B) The weight of tumour mass was scored after 25 days. C) After 25 days lung were explanted and the number of lung metastasis was counted in WT and homozygous mutant.

We reasoned that, if PlexinB1 would play a major role in this process, its ablation would impact on tumour angiogenesis, leading to reduced growth rate and metastatization of cancer cells. To test this, we exploited the well established B16 melanoma model, which is syngenic with the C57BL6 background of our mutants, and that we found to produce Sema4D (see Additional file 4). Melanoma cells were injected orthotopically in the subcute of WT, heterozygous and homozygous PlexinB1 mutant; tumour growth was followed over time, and tumour explants were eventually weighted at the end of the experiment after 25 days. We did not observe any significant difference between the slope of tumour growth curve or in the final weight of the tumours grown in WT or PlexinB1 null mice (Figure 8A, B). Moreover, we counted the number of lung metastases formed by tumours in normal and homozygous mutant mice and we did not observe significative differences (Figure 8C).

To scrutinize further vessel growth within the primary tumours, we stained tissue sections with an antibody against CD31 endothelial marker, and analyzed them by fluorescence microscopy. We could not reveal morphological differences between tumour vessels in mutant and WT mice. In addition, we quantified total vessel area and vessel density (the number of vessels per mm^2^) in tumour sections. In both cases, we did not measure a significant change in PlexinB1 -/- compared to WT mice.

## Discussion

Plexins were originally characterized for their role in the wiring of neural network as receptors for Semaphorin guidance cues. However, accumulating lines of evidence later prompted the investigators to reappraise and revise the biological role of these molecules. First, it was found that many Plexins are widely expressed outside the nervous system. Moreover, as the signal transduction mechanisms start to be uncovered, it is now clear that Plexins can impinge on a range of signaling pathways potentially regulating cell adhesion, cell proliferation and differentiation.

Here we demonstrate, by immunohistochemical analysis, the expression of PlexinB1 receptor and of its ligand Sema4D in diverse epithelial tissues and in the nervous system, suggesting that PlexinB1 might play multiple functional roles in vivo, during development and in the adult stage. In fact, this is the first extensive analysis, in the mouse embryo and in the adult, of PlexinB1 and Sema4D tissue distribution undertaken so far.

Previous expression studies and experiments in vitro had highlighted a potential role for secreted semaphorins and their receptors during tubular morphogenesis in the lung [22] and in the kidney [24], and it was hypothesized that these guidance cues may define permissive and restricted regions for cell migration. Interestingly, based on our expression analysis, Sema4D-PlexinB1 signaling might have a role in this function, for instance at the boundaries between different cell populations. Moreover, PlexinB1 can regulate the activation of Met [13], a tyrosine kinase receptor known to mediate tubular morphogenesis in fetal lung and kidney [25]. In spite of these facts, the genetic deletion of PlexinB1 did not lead to morphological or functional abnormalities, indicating that the function of this semaphorin receptor is redundant in development. Notably, developing kidney and lung also express PlexinB2 [20], another putative Sema4D receptor [26]. Although we observed that PlexinB2 binds to Sema4D with lower affinity than PlexinB1 (J.P. and L.T., unpublished observation), it is conceivable that PlexinB2 can compensate for the lack of PlexinB1 in these organs. In fact, by measuring PlexinB2 mRNA levels in tissues with real time PCR, we found significant expression in liver and kidney, which did not increase in PlexinB1-deficient mice (not shown).

These findings are in line with that found in previously reported mouse mutants of semaphorins or plexins expressed in developing lung (such as Sema3A [27], Sema3F [28], PlexinA1 [29]), which also lack to show obvious abnormalities of these organs [22,30]. Thus, this scenario suggests a high level of redundancy in semaphorin signaling during branching morphogenesis and in the maintenance of tissue homeostasis in lung and kidney. Moreover, since additional guidance cues are known to regulate these developmental processes, it is conceivable that the absence of semaphorin signals may be compensated by functionally related morphogenetic factors such as the Netrins and the Slits (for an extensive review see [31]).

In contrast to the lung and the kidney, PlexinB1 expression in the liver is not consistent with a function as receptor for Sema4D, since we could not detect significant expression of this semaphorin in the mouse liver at any stage. This may suggest that hepatocytes or other liver cells express as yet unidentified alternative ligands for PlexinB1. Furthermore, since it was shown that Plexins can associate in receptor complexes on the cell surface [13,14] or regulate homophilic cell-cell adhesion [32,33], it is also possible that PlexinB1 acts in the maintenance of liver tissue architecture by functionally regulating other receptors or homologous plexins.

It was previously shown that Sema4D-PlexinB1 signalling can collapse the growth cone of hippocampal neurons in culture [15]. Interestingly, we have observed loss of responsiveness to Sema4D in PlexinB1-deficient neurons derived from our mutants. On the other hand, our histological analysis revealed that the overall morphology, cell composition and neural connections in the hippocampus do not display obvious abnormalities in the absence of PlexinB1, suggesting a compensation of Sema4D function in development.

In the cerebellum, PlexinB1 is highly expressed in Purkinje (Pk) cells during the postnatal development. Notably, these neurons do not express PlexinB2 or CD72, which are lower affinity receptors for Sema4D [19,21]. Incidentally, it was shown that Pk cells express PlexinB3 [33], but we previously demonstrated that this receptor cannot bind Sema4D [34]. PlexinB1 distribution in the cerebellum appears consistent with the traditional role of Plexins as receptors for neurite outgrowth inhibitory cues. In fact, during the first postnatal days, Purkinje axons show exuberant sprouting of collateral branches that are eventually pruned during cerebellar development [23]. The final shaping of Pk axons is associated with the axonal myelination mediated by the olygodendrocytes that express Sema4D [21]. However, our analysis shows that Pk axons are correctly myelinated and the pruning of excessive branches occurs normally in the PlexinB1 mutant mice. It is noteworthy that PlexinB1 in Pk cells is also present in the perikaryon and dendrites. In addition, the protein shows different levels of expression in Pk cell subsets, corresponding to morphofunctional modules of the cerebellar cortex [35]. Together with the precise distribution of Sema4D in the molecular layer, these observations suggest that Sema4D-PlexinB1 signalling may play a role in regulating the patterning and plasticity of intracortical connectivity, although this may be compensated during cerebellar development by alternative pathways.

The absence of clear developmental defects in PlexinB1 mutants is consistent with that reported for Sema4D-deficient mice. In fact, the latter were only found to have minor defects in the immune response, likely attributed to defective CD72 signaling [36], which is an alternative low-affinity receptor for Sema4D expressed in lymphocytes.

Our expression analysis had revealed that PlexinB1 is importantly expressed in a number of adult tissues, potentially suggesting a physiological role beyond embryo development. To address this point, in addition to study tissue histology, we have performed blood tests to investigate major functions in PlexinB1-deficient mice. Our data show that liver and kidney functions were normal. In addition, we found that hematopoiesis and coagulation parameters were not affected (data not shown).

Furthermore, it was proposed that PlexinB1 expressed by endothelial cells may mediate the pro-angiogenic activity of Sema4D released by tumour cells [18]. Tumour-induced angiogenesis is of fundamental importance in cancer progression; in fact, hampering the formation of new vessels is now a popular strategy to restrain tumour growth. In order to test the functional requirement for PlexinB1 in this process, we exploited B16 melanoma transplants, which are commonly used to study tumour growth and angiogenesis in the context of syngenic C57BL/6 mouse models. We found that B16 cells produce Sema4D and tested their ability to grow and induce angiogenesis in PlexinB1-deficient mice. Our results suggest that the angiogenic activity of Sema4D may be mediated not only by PlexinB1, but also via other receptors expressed in endothelial cells (e.g. PlexinB2, see Additional file 4, panel C). Future studies will tell whether other tumour models are equally capable to grow in a PlexinB1-deficient tissue microenvironment.

## Conclusion

Collectively, our data concerning PlexinB1 and Sema4D expression suggest that PlexinB1 may carry out multiple functions during development and in the adult. Nonetheless, our analysis of PlexinB1 mutant mice did not show any major impairment in embryo development or differentiated functions in the adult, including the neo-angiogenetic response induced by tumour growth. We conclude that PlexinB1 plays a redundant role during development, in the maintenance of tissue homeostasis, and in tumour angiogenesis.

## Methods

### mRNA expression analysis

A human multiple tissue array (MTE™) was purchased from Clonetech laboratories (Cat. #775-1). It contains mRNA in amounts normalized for the expression of eight different housekeeping genes. The P32 radioactive labelled cDNA probe used for hybridization contained a 4 kb divergent sequence encoding plexin extracellular domain obtained by EcoRI restriction of human full length construct. The hybridisation was performed using Ultrahybe^® ^solution (Ambion Austin, TX, US) according to the protocol provided by the manufacturer.

Plexin expression in Human Umbelical Vein Endothelial Cells (Cambrex) was detected by RT-PCR using gene-specific primer pairs provided by Applied Biosystem (Hs00182227_m1 for PlexinB1, and Hs00367063_m1 for PlexinB2).

## mRNA In situ hybridyzation

Whole-mount *in situ *hybridization (ISH) was according to standard protocol. Briefly, embryos were fixed overnight at 4°C in 4% (w/v) paraformaldehyde in PBT (0.1% Tween 20 in PBS), progressively dehydrated in increasing concentrations of ethanol/PBT, stored at -20°C, then rehydrated in decreasing concentrations of ethanol/PBT and treated with proteinase K (10 μg/ml in PBS). They were then postfixed for 20 min in 4% paraformaldehyde, 0.1% glutaraldehyde, and 0.1% Tween 20 and prehybridized 1 hr at 70°C in 1.3× SSC, 50% formamide, 2% Tween 20, 5% dextran, 5 mM EDTA, and 50 μg/ml yeast RNA.

Sense and control antisense ryboprobes were synthesized using T7 and SP6 polymerases with a kit for DIG labeling (Roche Diagnostics). The probes were amplified by PCR (for PlexinB1: sense GCT AAC AGC TGT GGC AAT CA, antisense GGG ACA TTG GAA GCT ATG GA; for Sema4D sense GTC TTC GTC CTC AGG TCT GC, antisense CGA CAG GTT GAA GAT GAG CA) and subcloned in TOPO™TA vector (Invitrogen). Hybridization was performed overnight with DIG-labeled riboprobes in the same buffer of prehybridization. Washes with hybridization buffer were followed by RNase A treatment (10 μg/ml in 0.5 M NaCl, 10 mM Tris, pH 7.5, and 0.1% Tween 20, 1 hr at 37°C), and subsequent washes with hybridization buffer at 65°C. Embryos were then blocked in MABT (0.1 M maleate, 0.15 M NaCl, and 0.1% Tween 20, pH 7.5) containing 20% sheep serum, and incubated overnight at 4°C with anti-DIG-alkaline phosphatase (AP)-conjugate (Roche Diagnostics) diluted 1: 2000 in MABT with 2% sheep serum. After extensive washes with MABT, revelation was performed using nitro blue tetrazolium and 5-bromo-4-chloro-3-indolyl phosphate (Roche Diagnostics) in 0.1 M NaCl, 50 mM MgCl_2_, 0.1% Tween 20, and 0.1 M Tris pH 9.5.

### Histology and immunohistochemistry

Histology and immunohistochemistry were performed according to standard techniques. For the embryo and the epithelial tissues, samples were collected and fixed in 4% paraformaldehyde/PBS, embedded in paraffin and sectioned at 6 μm. Sections were deparaffinised and processed. For immunohistochemistry on paraffin sections, all primary antibodies were diluted in Tris buffered saline 0.1% Tween20, 5% FBS. Our anti-PlexinB1 rabbit polyclonal antibody (raised against the following epitope in the extracellular domain: RNLHLFQDGPGDNEC) was used 1:500; murine monoclonal anti-Sema4D (BD Transduction Laboratories, BD Biosciences, Palo Alto, CA) was used 1:100; anti Smooth muscle actin (Sigma) was diluted 1:500. Sections were incubated overnight with the primary antibody, washed, incubated for 1 hour with DakoCytomationEnVision+^® ^System (Dako, CA, US) and diamminobenzidine staining was developed according to manufacturer instructions. Haematoxylin, eosin and reagents for PAS staining were purchased from Bio Optica (Milan, Italy) and used following the instructions of the producer. Paraffin sections were examined with LeicaDMLB light microscope connected to a LeicaDFC320 digital camera.

For the cerebellum the histology was performed as previously described [37]. Briefly, deeply anaesthetized mice were transcardially perfused with 4% paraformaldehyde in 0.12 M phosphate buffer (pH 7.2). The brains were dissected, kept in the same fixative overnight and cyroprotected in 30% sucrose 0.12 M phosphate buffer. The cerebella were cut by freezing microtome in 30 μm thick sagittal or frontal sections and collected in Tris buffered saline (pH 7.4). Anti-Calbindin D-28K (CaBP; rabbit polyclonal; Swant, Bellinzona, Swizerland) was used 1:3000; anti-Myelin basic protein (MBP; mouse monoclonal; Sternberger monoclonal, Baltimore, MD, USA) was diluted 1:2000; anti-Parvalbumin (mouse monoclonal, Swant, Bellinzona, Swizerland) was diluted 1:2000; anti PlexinB1 monoclonal antibody was used 1:500. The antibodies were diluted in phosphate buffered saline (PBS) with 0.25% Triton X-100 with 0.2% normal serum of the species of the secondary antibody. For immunohistochemistry sections were incubated for 1 h with biotinylated secondary antibody (1:200; Vector Laboratories, Burlingame, CA, USA) reacted with the avidin-biotin-peroxidase method (Vectastain, ABC standard Kit; Vector Laboratories, Burlingame, CA, USA) and revealed using diamminobenzidine as a chromogen. For double immunofluorescence sections were incubated for 1 h with FITCH conjugated anti-rabbit antibody (1:200 in PBS-triton with 0.2% normal serum; Sigma). After rinsing, they were again incubated overnight at 4° biotinylated anti-mouse antibody (1:200; Vector Laboratories, Burlingame, CA, USA), followed by Texas Red conjugated streptavidin (1:200 in PBS-Triton; Molecular Probes, Eugene, OR, USA). Nissl staining was performed according to ordinary techniques. Cerebellar sections were examined with a Zeiss Axiophot light microscope and an Olympus Fluoview 300 confocal microscope.

Digital images were processed with Adobe Photoshop 6.0 to adjust contrast.

The number of glomeruli in the kidney was counted in sagittal sections taken in the middle of the kidney and counterstained with haematoxylin and eosin. Five sections were evaluated per mouse and three wild type mice were compared with three mutant mice.

For vessel staining, tumour samples of similar volume (about 5 mm in diameter) were snap frozen in isopropanol at -60°, sectioned at the criostat and postfixed for 10' in 4% PFA. Sections were incubated with anti-CD31 (1:200 BD Pharmingen, USA) overnight, washed and incubated with anti-Rat Alexa 546 (1:500 Molecular Probes, USA). To reveal Sema4D expression in tumour samples by immunohistochemistry, tissue sections were post-fixed with acetone and incubated with monoclonal antibody clone BMA-12 (eBioscience), diluted 1:400.

Pictures were taken with a LeicaDM IRB microscope connected to a Leica DC350FX camera and analyzed with Metamorph 6.3 software (Molecular devices, USA).

### Generation of PlexinB1 mutant mice

A cosmidic clone containing the murine PlexinB1 gene was obtained by screening a RZPD (Berlin, Germany) genomic library. A targeting vector was designed to flank exons 22 and 23 of PlexinB1 with two loxP sites. A neo gene flanked by two FRT sites and a LacZ reporter gene were placed in frame with exon 24. A 10 kb fragment was used as a 5' homology region, a 1 kb fragment was placed between the two loxP sites, and a 2 kb fragment was used as 3' homology region. Embryonic stem cells (W9.5) were transfected, cultured and selected for resistance against G418. Homologous recombined clones were identified by Southern blot analysis of EcoRI-digested DNA, using an external probe that was amplified by PCR (sense primer GGT CTG ACC CTG GAT ATG GA; antinsense primer CCA CCCTCT TTT ATG CCT GA). Chimeric mice were generated by injection of homologous recombined ES cell clones into blastocystes from C57BL/6 mice. Matings of male chimeras to C57BL/6 females yielded germline transmitted offsprings. Mice carrying the mutated PlexinB1 gene were crossed with C57BL/6 CRE deleter mice in order to achieve the excision of exons 22 and 23. The correct excision was checked by PCR (sense AAC ACC ATG TGT ATG CTG GAG AGG TCA GGG; antisense CAT CGC CTT CTA TCG CCT TCT TGA CG). Mice genotyping was carried out by PCR using a primer pair for the WT (sense AAC ACC ATG TGT ATG CTG GAG AGG TCA GGG; antisense GGG TCA CTG ATT CGT TTC TCA GAA CAC TGA C) and the same primers used to check CRE excision for the mutant allele.

Loss of PlexinB1 expression was confirmed in mutant mice by protein immunopurification and Western blotting using IC-2 antibodies, as previously described [38].

### Cultures of hippocampal neurons

Primary hippocampal neurons, isolated from neonatal P1 mice, were cultured as previously described [39]. Briefly, the hippocampi were isolated, trypsinized and cultured at low density in Neurobasal Medium supplemented with 2% B27 and 2% FCS. After 48 hours in culture neurons were stimulated for 1 hour with a purified preparation of Sema4D. Soluble Sema4D was purified from the conditioned media of cells transfected with a cDNA expression construct, generated by recombinant PCR as previously described [40].

### Tumourigenesis assays in vivo

Sema4D expression in melanoma cells was detected using clone 30 antibody (BD-Transduction lab), by immunopurification and Western blotting experiments according to standard protocols [38]. Tumourigenesis assays were performed as previously described [41]. Shortly, 75000 B16 melanoma cells were diluted in PBS up to a final volume of 200 μl and injected subcutaneously into the right posterior flanks of 8-week old WT, heterozygous and homozygous PlexinB1 mutant mice. Tumour volume was calculated as described [41]. At the end of the observation period, tumours were weighed. Superficial pulmonary metastases were counted under a stereoscopic microscope taking advantage of the black pigmentation of B16 melanoma cell line.

### Blood tests

Blood tests were performed according to ordinary analytical techniques. Briefly, blood samples were collected via intra-cardiac puncture in presence of Na Citrate to avoid blood clotting and analyzed using photometric method (Modular Roche diagnostic).

## Abbreviations

Pk Purkinje, WT wild type, MBP myelin basic protein

## Authors' contributions

PF carried out the expression analysis by in situ hybridization and by immunohistochemistry, generated the targeting vector, screened the recombinant ES cell clones, analyzed PlexinB1 mutants, performed the tumorigenic assays and drafted the paper. JP carried out part of the genotyping and checked for PlexinB1 expression in mutant mice by western blot. SG helped in the study of PlexinB1 and Sema4D expression in the cerebellum. FR participated at the analysis of CNS of mutant mice and in the writing of the manuscript. BJE supervised the axonal collapse assays and revised the manuscript. FM helped in the whole mount in situ hybridization and in the experimental design of the targeting construct. LA produced recombinant ES cell and started the production of chimeric mice. AS performed the analysis of blood samples. PMC participated at the experimental design. RAF supervised the production of recombinant ES cells and chimeric mice. LT designed and coordinated the study, and revised the manuscript.

**Figure 9:**
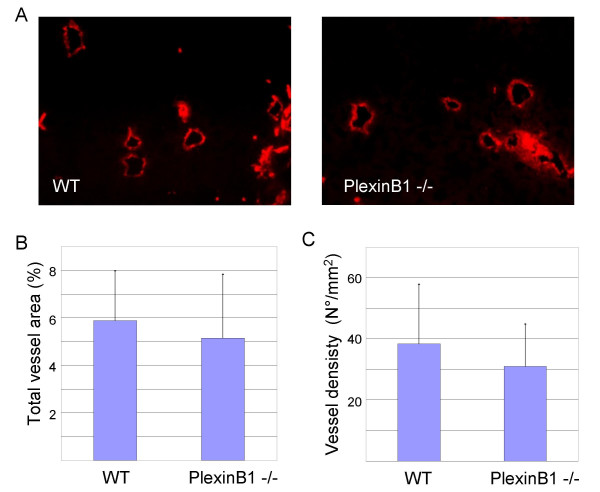
**Tumour angiogenesis in PlexinB1 mutant mice**. A) Representative images of tumour vessels in WT and PlexinB1 -/- mice stained for CD31 endothelial marker. B) Total vessel area expressed as percentage of the tumour section occupied by vascular tissue. C) Vessel density expressed in number of vessels per mm^2^.

## Supplementary Material

Additional file 1 **Whole mount mRNA expression at E12**. PlexinB1 (A, B) and Sema4D (C, D) display at E12 an overlapping expression pattern in diverse sensory structures, like in the dorsal root ganglia (black arrow) and in the trigeminal ganglion (red arrow). Moreover, PlexinB1 and Sema4D mRNA are found in the otic vesicle (green arrow). Click here for file

Additional file 2 **Anti-PlexinB1 antibody**. The PlexinB1 immunoreactivity in the adult liver and in the embryonic lung (A and C respectively) is blocked by the competition with the immunogenic peptide used to arise the anti PlexinB1 antibody (B and D). Click here for file

Additional file 3 **Hippocampus histology in WT and PlexinB1 mutants**. The comparison between the hippocampi of WT (A, B and C) and PlexinB1 mutant mice (D, E and F) did not reveal overt abnormalities of the cytoarchitectonics and cellular composition in the latter. The overall morphology is shown by Nissl staining in A and D. Anti-Calbindin (B and E) and anti-Parvalbumin (C and F) staining show that mutant hippocampi have normal complement of interneurons and typically patterned connections. Cornu Ammonis CA; Dentate Gyrus DG. Click here for file

Additional file 4 **Sema4D expression in B16 melanoma cells**. A) Sema4D expression is detected in mouse B16 melanoma cells by immunopurification followed by immunoblotting with a specific antibody (MoAb, clone 30). A murine tumour cell line that does not express the semaphorin, mammary carcinoma 66cl4, provided a specificity control. Lysates of human leukemia cells Jurkat, known to over-express and release Sema4D in secreted form, were included as positive control. Vinculin was detected in total protein lysates to provide a loading standard (at the bottom). B) The expression of Sema4D in B16 tumours growing in mice was furthermore detected in situ in tissue sections by immuno-histochemistry, using MoAb clone BMA-12. C) HUVEC (HU) endothelial cells express both PlexinB1 and PlexinB2, as demonstrated by semi-quantitative RT-PCR. Human carcinoma cells SKBR3 (SK) provided a positive control for plexin expression. Control reactions contained everything but cDNA. Click here for file
