# Supplementary figures and images for: Plexin-B1 plays a redundant role during mouse development and in tumour angiogenesis
Source: BMC Dev Biol. 2007 May 22;7:55. doi: 10.1186/1471-213X-7-55 (PMC1890291; doi:10.1186/1471-213X-7-55)

## Slide 1
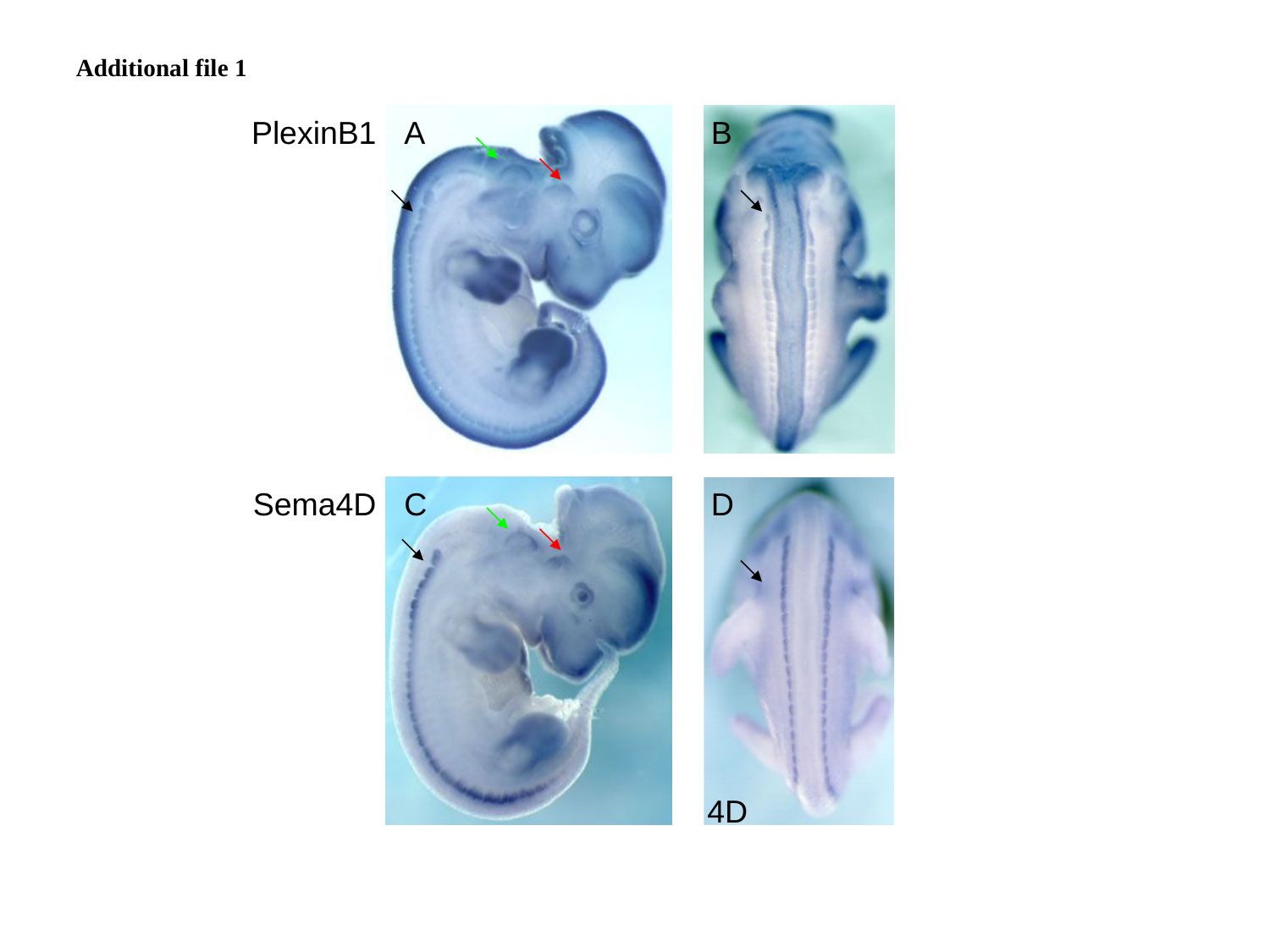

Additional file 1
A
B
PlexinB1
Sema4D
C
D
4D

Supplement: Additional file 1 — Whole mount mRNA expression at E12. PlexinB1 (A, B) and Sema4D (C, D) display at E12 an overlapping expression pattern in diverse sensory structures, like in the dorsal root ganglia (black arrow) and in the trigeminal ganglion (red arrow). Moreover, PlexinB1 and Sema4D mRNA are found in the otic vesicle (green arrow). [file 1471-213X-7-55-S1.ppt]

## Slide 1
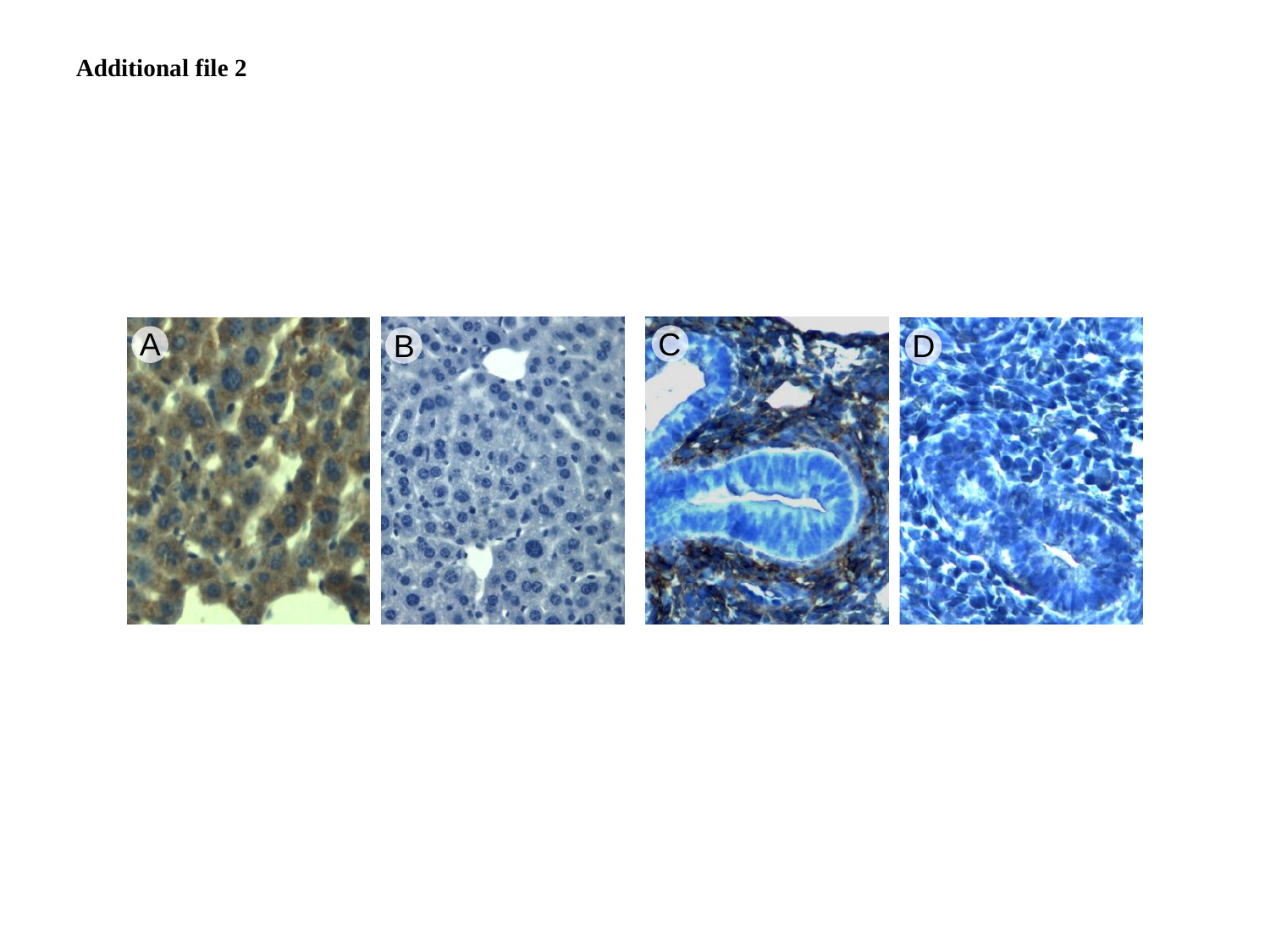

Additional file 2
A
C
B
D

Supplement: Additional file 2 — Anti-PlexinB1 antibody. The PlexinB1 immunoreactivity in the adult liver and in the embryonic lung (A and C respectively) is blocked by the competition with the immunogenic peptide used to arise the anti PlexinB1 antibody (B and D). [file 1471-213X-7-55-S2.ppt]

## Slide 1
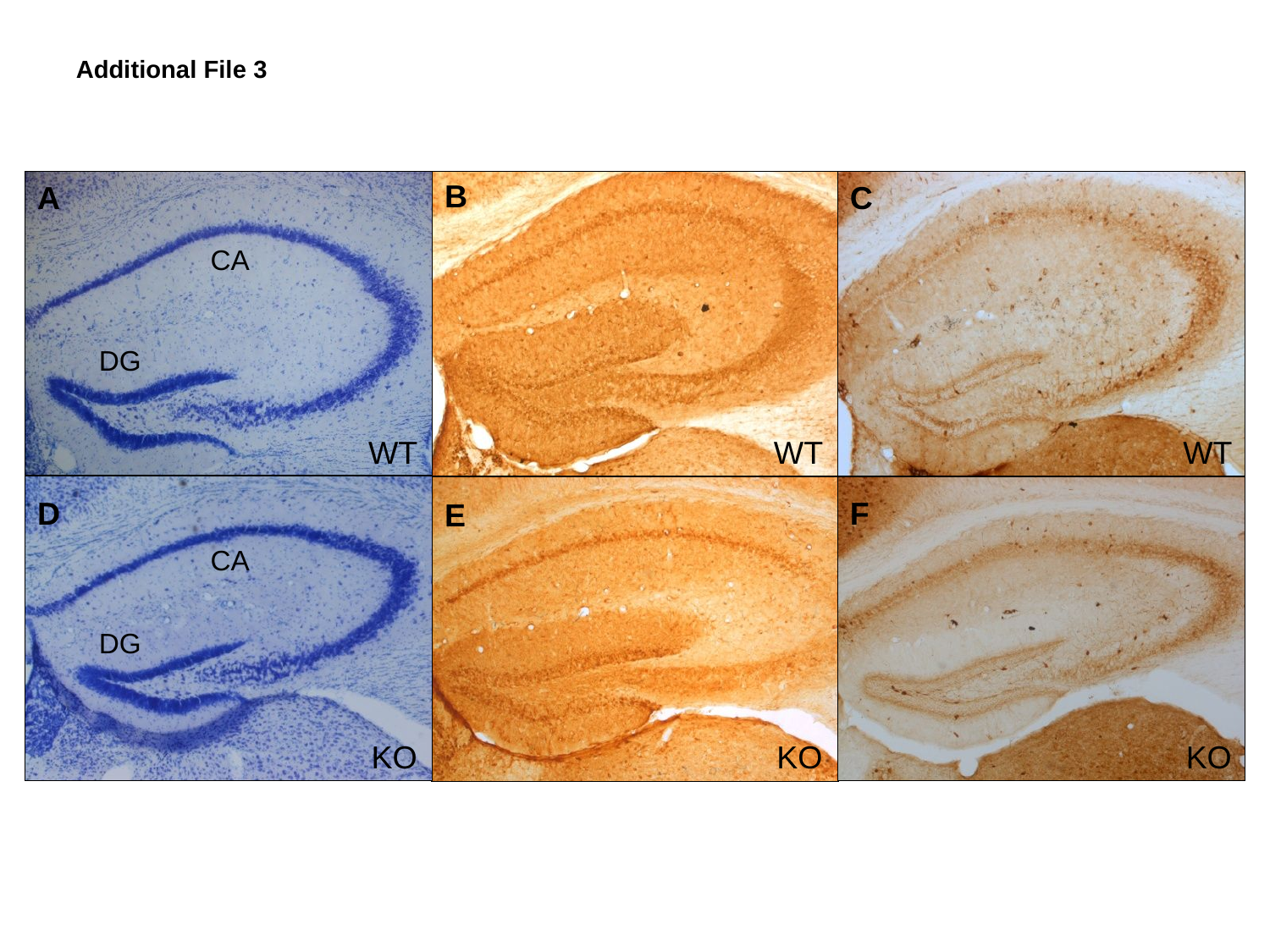

Additional File 3
B
A
C
CA
DG
WT
WT
WT
D
F
E
CA
DG
KO
KO
KO

Supplement: Additional file 3 — Hippocampus histology in WT and PlexinB1 mutants. The comparison between the hippocampi of WT (A, B and C) and PlexinB1 mutant mice (D, E and F) did not reveal overt abnormalities of the cytoarchitectonics and cellular composition in the latter. The overall morphology is shown by Nissl staining in A and D. Anti-Calbindin (B and E) and anti-Parvalbumin (C and F) staining show that mutant hippocampi have normal complement of interneurons and typically patterned connections. Cornu Ammonis CA; Dentate Gyrus DG. [file 1471-213X-7-55-S3.ppt]
