# Supplementary material for: Plexin-B1 plays a redundant role during mouse development and in tumour angiogenesis
Source: BMC Dev Biol. 2007 May 22;7:55. doi: 10.1186/1471-213X-7-55 (PMC1890291; doi:10.1186/1471-213X-7-55)
Supplement: Additional file 4 — Sema4D expression in B16 melanoma cells. A) Sema4D expression is detected in mouse B16 melanoma cells by immunopurification followed by immunoblotting with a specific antibody (MoAb, clone 30). A murine tumour cell line that does not express the semaphorin, mammary carcinoma 66cl4, provided a specificity control. Lysates of human leukemia cells Jurkat, known to over-express and release Sema4D in secreted form, were included as positive control. Vinculin was detected in total protein lysates to provide a loading standard (at the bottom). B) The expression of Sema4D in B16 tumours growing in mice was furthermore detected in situ in tissue sections by immuno-histochemistry, using MoAb clone BMA-12. C) HUVEC (HU) endothelial cells express both PlexinB1 and PlexinB2, as demonstrated by semi-quantitative RT-PCR. Human carcinoma cells SKBR3 (SK) provided a positive control for plexin expression. Control reactions contained everything but cDNA. [file 1471-213X-7-55-S4.ppt]

## Slide 1
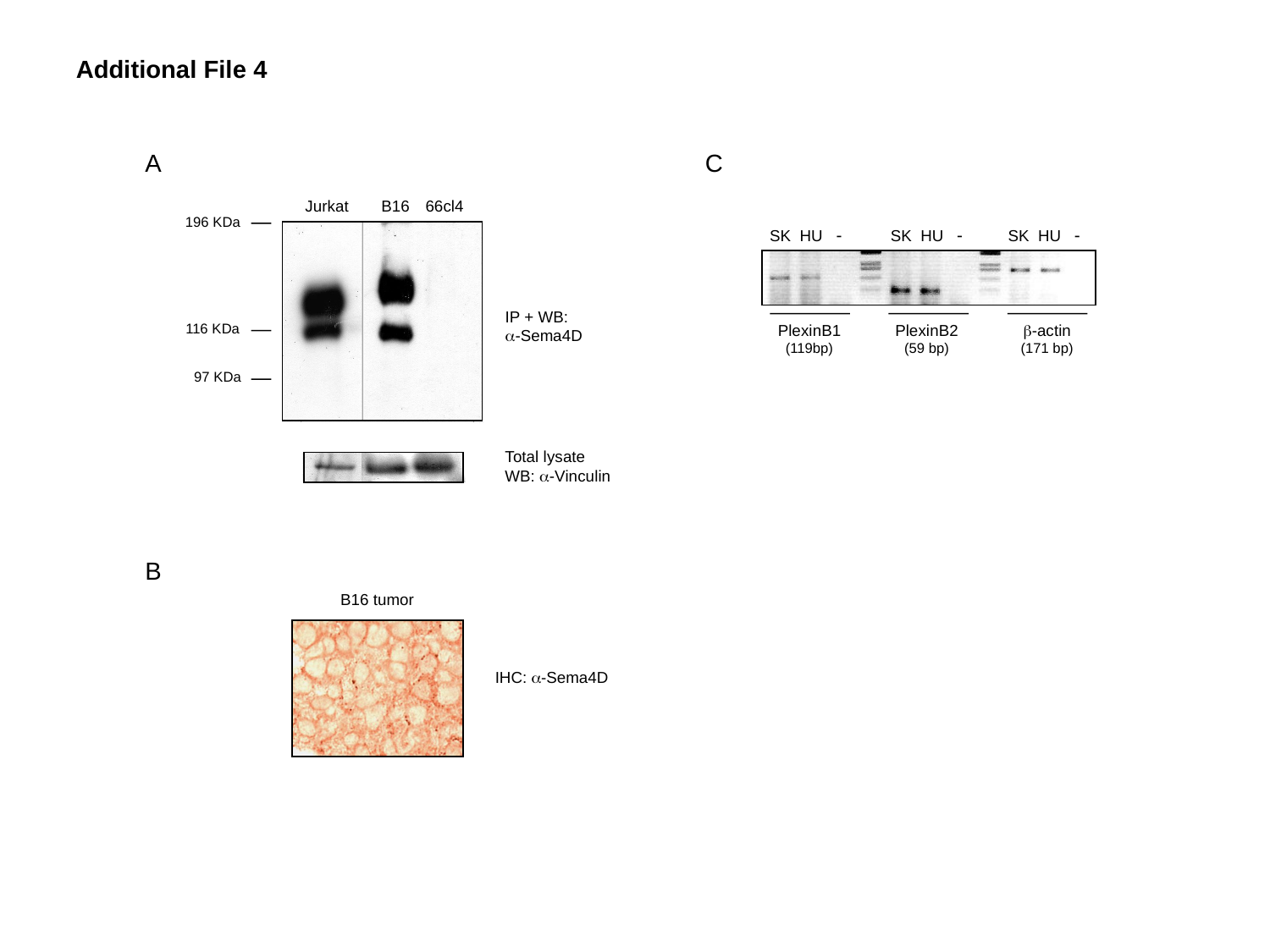

Additional File 4
A
C
Jurkat
B16
66cl4
196 KDa
SK HU -
SK HU -
SK HU -
IP + WB:
α-Sema4D
116 KDa
PlexinB1
(119bp)
PlexinB2
(59 bp)
β-actin
(171 bp)
97 KDa
Total lysate
WB: α-Vinculin
B
B16 tumor
IHC: α-Sema4D
